# Celiac Disease—Narrative Review on Progress in Celiac Disease

**DOI:** 10.3390/foods14060959

**Published:** 2025-03-11

**Authors:** Marek K. Kowalski, Danuta Domżał-Magrowska, Ewa Małecka-Wojciesko

**Affiliations:** Department of Digestive Tract Diseases, Norbert Barlicki University Hospital, 90-153 Lodz, Poland; mekamed14@gmail.com (M.K.K.); danuta.magrowska@gmail.com (D.D.-M.)

**Keywords:** celiac disease, diet-resistant celiac disease, specific antibodies, HLA DQ2 and DQ8, enteropathy

## Abstract

Celiac disease is defined as a systemic immunological disorder caused by gluten (gliadin and other prolamin) in genetically predisposed individuals, who present with a variety of gluten-dependent symptoms, specific antibodies, the presence of the HLA DQ2 and DQ8 histocompatibility antigen, and enteropathy. Its prevalence, depending on the studied population and methodology, is estimated at 0.75–1.6% of the general population. During the complex immune reaction it induces, most cells involved in inflammatory processes are activated, which leads to the gradual atrophy of intestinal villi and the proliferation of enterocytes within intestinal crypts. The pathogenesis of celiac disease is extremely complicated and is still the subject of research. According to the current diagnostic guidelines, the following criteria should be taken into account: clinical symptoms (intestinal and extraintestinal), the presence of antibodies against tissue transglutaminase in the IgA class, the level of total IgA, and the presence of typical histological changes in duodenal biopsies. Diet-resistant celiac disease is one of the most important clinical challenges, causing serious complications. Currently, the basic method for treating celiac disease is an elimination diet (i.e., the exclusion of products that may contain gluten from the diet), however, new therapeutic strategies are still being sought, mainly based on supplementation with exogenous endopeptidases, modification of the immune response, and the use of zonulin inhibitors and transglutaminase 2 inhibitors. Clinical trials of new drugs are ongoing. The gradually expanding knowledge about the pathogenesis of celiac disease may allow for the development of new therapeutic strategies for both patients with a mild disease course, as well as those that are diet-resistant.

## 1. Introduction

Celiac disease (CD) is defined as a systemic autoimmune disorder induced by cereal prolamins in genetically predisposed individuals, who experience a variety of gluten-dependent symptoms, specific antibodies, the presence of HLA-DQ2 and DQ8 histocompatibility antigens, and enteropathy [1,2]. Characteristic intestinal symptoms of celiac disease may include diarrhea (13–96%), abdominal pain (8–90%), vomiting (26–33%), flatulence (5–10%), and fatty stools [3,4,5,6]. There are also extraintestinal symptoms that are related to gastrointestinal dysfunction, mainly in the course of malabsorption, ultimately leading to numerous disorders affecting most body systems.

Celiac disease, untreated for years, can also lead to the development of malignant tumors, such as esophageal cancer, small intestine cancer, and lymphoma, especially T-cell lymphoma, which occurs mainly in the small intestine. These cancers are rare, but they occur significantly more often in patients with celiac disease than in the general population [7].

Patients with celiac disease also suffer from other comorbidities—the most prevalent are autoimmune diseases such as type 1 diabetes (7%), Duhring’s disease (3%), and thyroid diseases (5–21%) [8,9], but also neuropsychiatric disorders (3.9–35.9%) [10].

In this publication, we would like to present the progress in knowledge on the pathogenesis of celiac disease, the latest diagnostics novel management strategies, and treatment monitoring.

### 1.1. Classification

Chronic inflammation in celiac disease leads to gradual changes in the duodenal mucosa. The histopathological picture of the small intestinal mucosa was described and systematized initially by Marsh et al. and then modified by Oberhuber et al. [11,12,13,14,15] According to current classification, the histopathological changes in the small intestinal mucosa are divided into five types. In type 0 (preinfiltrative), the mucosal picture is normal and the ratio of intraepithelial lymphocytes (IELs) to enterocytes is less than 30. In type 1 (infiltrative stage), an increase in the number of intraepithelial lymphocytes (IELs) above 30 per 100 enterocytes is observed, without other changes. In type 2 (infiltrative–hyperplastic), in addition to an increased number of IELs, intestinal crypt hyperplasia and an elevated mitotic index are observed, with a normal villi structure. Within type 3 (flat destructive), three subtypes are distinguished. In subtype 3a (progressive villous atrophy), in addition to the changes characteristic of type 2, there also comes mild villous atrophy. In subtype 3b (almost complete shortening of the villi), villous atrophy is clearly marked, with crypt hyperplasia and a number of IELs > 30/100 enterocytes, and in 3c (complete villous atrophy), the villi are flat, with crypt hyperplasia and a number of IELs > 30/100 enterocytes. Type 4, hypoplastic–atrophic, involves complete villous atrophy with a normal crypt structure and normal number of IELs [11,12,13,14,15].

According to the current Oslo definitions for celiac disease, depending on the clinical picture and additional tests, several forms of celiac disease have been identified, as follows: typical, atypical, asymptomatic, potential, and refractory [16,17]. Typical celiac disease is diagnosed based on intestinal symptoms (such as weight loss, chronic diarrhea, abdominal pain, vomiting, flatulence, and steatorrhea), Marsh 3 villus atrophy, the presence of characteristic antibodies, and the presence of HLA-DQ 2 or DQ8. Atypical celiac disease differs from overt celiac disease by the presence of extraintestinal symptoms and the absence of classical symptoms. The most frequent extraintestinal symptoms are anemia (3–30%), iron deficiency anemia (40%), folic acid deficiency (20%), and vitamin B12 deficiency (17%). Among celiac disease patients, deficiencies in the fat-soluble vitamins A, D, K, and E and elevated levels of aminotransferases are observed more frequently than in the general population [18]. Osteopenia (54%) and osteoporosis (12%), mainly due to a vitamin D deficiency (34%), and hypocalcemia leading to tetany are among the most common complications, which may present as the first symptoms of the disease [19,20,21,22,23,24]. A meta-analysis of 26 studies showed lower levels of vitamin D among celiac disease patients who did not adhere to a gluten-free diet than in the general population. At the same time, it was found that, after the introduction of a gluten-free diet, vitamin D levels were elevated [25]. It should be remembered that vitamin D deficiency is also a common phenomenon [26] among residents of countries with a high sun exposure [27]. Zanchetta et al. reported that bone loss is observed in about 50% of patients with CD [23,28] (Table 1). In another study by Bai et al., osteopenia incidence in CD was even higher—84% [21]. Galli et al., Ganji et al., Walker et al., and Sayar et al. estimated osteopenia frequency to be between 32 and 67% in all CD patients. Osteoporosis is less frequent and, among CD patients, yields from about 11 to 38% [29,30,31,32,33] (Table 1). In the newest study, Skoracka et al. revealed that women with CD have a decreased bone mass density (BMD) and anthropometric parameters such as body mass, BMI, fat tissue, muscle mass, and fat-free mass [34] (Table 1). There are also many neurological diseases associated with celiac disease, like gluten-dependent cerebellar ataxia, progressive cerebellar ataxia, spinocerebellar degeneration, increased epileptic activity on EEG recording and epilepsy, cerebral calcifications with convulsions, restless legs syndrome, myopathy, and peripheral neuropathy in the course of vitamin B1 and B12 deficiency, as well as dementia [35,36,37,38]. Patients often suffer from mental illnesses, especially schizophrenia and depression, as well as autism [39,40,41,42,43].

The clinical manifestation of pediatric patients with celiac disease has changed over the years. Nowadays, children present many different symptoms in the gastrointestinal tract, such as diarrhea, bloating, and abdominal pain, but also constipation, gastroesophageal reflux disease, vomiting, and dyspepsia. They also present extraintestinal symptoms like malabsorption syndrome, failure to thrive, short stature, dental enamel defects, delayed puberty, and joint pain or anemia due to isolated iron deficiency [44,45]. In children, there are more frequent neuropsychiatric symptoms, like mood changes, learning disabilities, confusion, fatigue, memory loss, depression, persecutory delusions, and psychosis [46,47].

In asymptomatic celiac disease, only the presence of serum anti-tTG, -EMA, and -DPG antibodies and villous atrophy detected in duodenal bioptates are observed. Potential celiac disease is characterized by the presence of characteristic HLA and elevated levels of anti-tTG, -EMA, and -DGP antibodies, but not enteropathy. It concerns individuals who have shown symptoms of gluten-sensitive enteropathy in the past. It should be noted that patients with potential celiac disease are at risk for villus atrophy [2,48]. Refractory celiac disease (RCD) occurs when a patient, despite adherence to a strict gluten-free diet for 12 months, does not achieve villous regeneration [49,50,51]. The two following forms of RCD are distinguished: type I, in which in a histopathological examination, activated T cells constitute up to 20% of all those visible in the preparation, and type II, when their presence is higher [52,53,54]. RCD accounts for 0.04–1.5% of all celiac disease cases and is mainly observed in patients diagnosed over the age of 50 [55,56]. In spite of research progress, RCD represents the important clinical challenge and its management is difficult.

Once a correct diagnosis has been made, in accordance with the guidelines for patients with celiac disease, serologic surveillance is recommended every 3–6 months for the first year after diagnosis and then every 1–2 years. It has been considered that a lack of normalization of antibody levels within a period of 12 months indicates gluten contamination of consumed food or RCD [2,49,51,57,58,59,60,61,62,63].

### 1.2. Epidemiology

**Figure 1:**
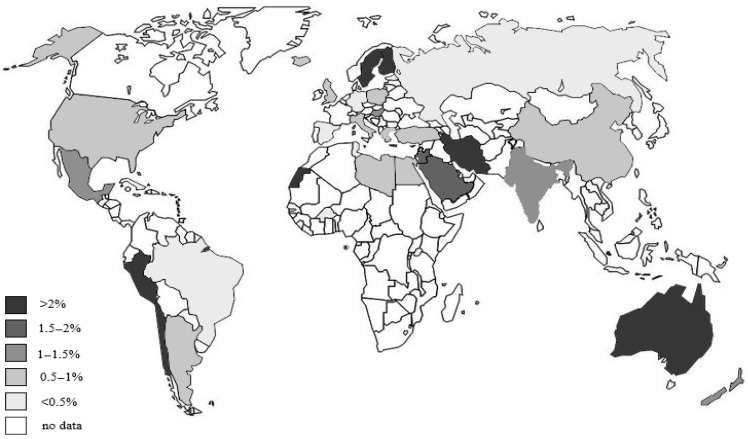
World map of celiac disease prevalence.

Celiac disease is considered one of the most common autoimmune diseases. Its prevalence, depending on the investigated population and methodology, is estimated at 0.28–5.6% of all screened people [16,64,65,66]. When analyzing the distribution of celiac disease incidence, there is no clear geographical latitude or continent where celiac disease occurs more often (Figure 1) [65,66,67,68,69,70,71,72,73,74,75,76,77,78,79,80,81,82,83,84,85,86,87,88,89]. A detailed analysis conducted among the inhabitants of the United States of America showed that celiac disease occurs significantly more frequently in Caucasian people (1.01%) than in African American (0.2%) or Latino (0.3%) people [67].

Celiac disease develops in genetically predisposed subjects. It has been shown to occur more frequently in relatives of celiac patients than in the general population. The risk of developing celiac disease was 4.5–7.5% among first-degree relatives and 2.3–2.6% among second-degree relatives. It was diagnosed more often in women than in men who were first-degree relatives of celiac patients (8.4% vs. 5.2%) [68,69,70].

Studies conducted around the world have observed considerable geographic variations in the prevalence of celiac disease. It occurs mainly in Europe (1.5–2.4% in Finland, 0.7% in Italy, and 0.3% in Germany [71]), North Africa (0.28–5.6%), the Middle East (1.9–2%), North America (0.75%), and India. It is much less frequent in Australia (0.3%). It is equally rare in Asia, excluding India, as well as in Central and South Africa (0.4–0.6%) (Table 2) [72,73,74,75,76,77].

Most population studies are based on an analysis of the frequency of increased serum anti-tTG antibodies, which does not fully correspond to the frequency of celiac disease [78]. Many studies assessing the incidence of celiac disease are conducted among patients with type 1 diabetes [74].

### 1.3. Etiopathogenesis

Celiac disease develops due to an abnormal immune response to ingested prolamin contained in cereals such as rye, wheat, barley, and oats, which are, respectively, gliadin, secalin, hordein, or avenin, in genetically predisposed individuals with specific histocompatibility antigens (HLA-DQ2 or HLA-DQ8) [90]. Gluten proteins are a heterogeneous group consisting of high-molecular-weight (HMW) and low-molecular-weight (LMW) subunits. Although oats contain prolamins that are toxic to some patients with CD, pure oats are not contraindicated in patients with celiac disease in some high-income countries. However, because of this controversy, their use should be associated with more careful monitoring of the patient [91,92,93]. A meta-analysis of 433 studies examining the immunogenicity of oats did not show any effect of their consumption on the health of patients with celiac disease [94]. Also, a double-blind study on a group of 177 patients with confirmed celiac disease, lasting 15 months, did not reveal an increase in antibody levels and did not cause symptoms or an increased intestinal permeability [95]. Perhaps this is the reason that ESPGHAN, WGO, ECD, NICE, BCG, and ACG guidelines allow the use of oats in CD [2,49,57,59,60,61,63]. On the other hand, the presence of t-cell lymphocytes with specific DQ 2.5-ave-1a-c and DQ 2.5-ave-2 receptors has been demonstrated [96]. In addition, Russian guidelines categorically exclude the possibility of using refined oats in a gluten-free diet [58].

After ingesting gluten-rich cereals, gluten is partially broken down in the stomach by pepsin. The long-chain protein fragments obtained in this way, after contact with the gastrointestinal mucosa, show significant immunogenicity, causing an increase in the concentration of inflammatory interleukins in the blood [97].

Prolamin degradation products are further digested in the duodenum and small intestine with prolyl oligopeptidase [98]. Gluten treated by endopeptidases is broken down into short glutamine and proline-rich peptide chains. The level of degradation resulting from the digestive process depends on the type of the contained protein. Protein digestion results in the formation of oligopeptides which are then deamidated by tissue transglutaminase and stimulate the activity of T lymphocytes [99,100,101].

The intestinal microbiota play an active role in the process of gluten protein digestion. In celiac patients, especially those not complying with a gluten-free diet, the presence of a greater number of fermenting bacteria in the gut has been demonstrated [102]. It should also be noted that the introduction of a gluten-free diet leads to an increase in the number of *Enterobacteriaceae* and *Escherichia coli* with a simultaneous reduction in the number of *Bifidobacter* sp., both in celiac patients and in healthy individuals. In Sanz and De Palma’s study, ten healthy subjects followed a gluten-free diet for a month. After this period, fecal samples revealed significantly reduced counts of *Bifidobacterium* and *Lactobacillus* and increased counts of *Prevotella*, *Clostridium*, *Enterobacteriaceae*, and *E. coli* [103,104]. The same microflora changes were observed in patients both with celiac disease strictly adhering to the diet and in patients with untreated celiac disease. A reduction in the number of probiotic strains and the promotion of non-beneficial strains can lead to numerous gastrointestinal complaints such as flatulence, abdominal pain, empty belching, and even fever and diarrhea [105,106,107]. Many different oral and intestinal bacteria produce their own endopeptidases and hydrolases that contribute to the digestion of protein chains resulting from the degradation of gliadin. Therefore, excluding gluten from the diet may promote other bacterial strains, leading to adverse changes in the intestinal flora described above [108,109,110,111,112,113].

Fragments of undigested proline-rich proteins in contact with the intestinal mucosa increase in IL-15, which, in turn, stimulates activated T and NK cells to produce chemokines and proinflammatory cytokines [114,115,116]. Moreover, IL-15 promotes NK cell apoptosis [117,118]. This process is additionally intensified by a direct contact reaction between gliadin fraction (p31–43) and the intestinal mucosa, leading to enterocyte cytoskeleton reconstruction and, subsequently, to apoptosis [119,120,121] Furthermore, fragments of partially digested gliadin have been shown to bind to CD95/FAS receptors belonging to the group of TNF-alpha (Tumor Necrosis Factor alpha) family receptors, leading to apoptosis by activating caspase 8 and 10 [122].

Moreover, an increased intestinal mucosal permeability is caused by the loosening of the tight junctions between enterocytes [123] The pathomechanism of this phenomenon was described by Fasano et al. They showed that gliadin fragments (111–130 and 151–170) in the small intestine bind to the CXCR3 receptor [124]. This leads to the release of zonulin (a protein similar to the toxin produced by cholera bacillus) from enterocytes of the small intestinal mucosa [125,126,127]. This protein binds to the protease-activating receptor (PAR2). As a result, the tight junctions between enterocytes are disrupted and transepithelial electrical resistance is reduced, leading to increased intestinal wall permeability [128,129,130,131]. Both described processes are responsible for the increased penetration of undigested short-chain proline-rich proteins into the submucosa. At this stage, the intestinal bacterial flora may also play an important role. In untreated celiac patients, B. lactis and L. fermentum have been shown to increase intestinal mucosal permeability by reducing transepithelial resistance and increasing zonulin expression [132].

In vitro studies have demonstrated that gliadin fragments (mainly p62–75 and p57–68) undergo tissue transglutaminase-mediated deamidation. The deamination process has been shown to be necessary to trigger further immune responses [133,134,135]. In the next step, deamidated gliadin peptides bind with DQ2 or DQ8 receptors expressed on antigen-presenting cells (APCs) [136,137,138]. Two heterodimers, DQ2 (DQ2.2 and DQ 2.5) and DQ8, participate in the antigen presentation process [139,140,141,142]. As a result of the reaction, HLA-DQ2 and DQ8 are complexed with deamidated α-gliadin fragments [143]. The protein fragments are then presented to B lymphocytes by APCs, initiating further humoral response.

The existence of molecular mimicry resulting from a similar three-dimensional appearance of proteins, mainly tissue transglutaminase and specific gliadin fragments, has been proven [144]. As a result, B cells produce antibodies against both tissue transglutaminase and gliadin and deamidated gliadin peptides [145,146,147,148]. It should be noted that, under the influence of a number of inflammatory cytokines produced by monocytes and T cells, such as INFα, IL-15, IL-18, and IL-21, the inflammatory response in the course of celiac disease is directed towards the Th1-dependent inflammatory pathway [149,150,151,152,153]. At the same time, INF γ and TGF α stimulate monocytes and myofibroblasts, damaging the enterocyte stroma by producing matrix metalloproteinases (MMP-1, MMP-3, and MMP-12). This leads directly to the intensification of the apoptosis process and to increased mucosal permeability [154,155,156].

The above-mentioned CXCR3 receptor present on enterocytes also interacts with fragments of gliadin (p261–277 and p270–286), leading to an increase in the concentration of inflammatory interleukins, especially IL-8, a neutrophil chemokine. This reaction was observed only in celiac patients and persisted despite a gluten-free diet [157].

On the other hand, the contact of gliadin fragments with enterocytes leads to an increased production of EGF (epidermal growth factor), which stimulates crypt enterocyte proliferation, leading to their hyperplasia [158,159].

**Figure 2:**
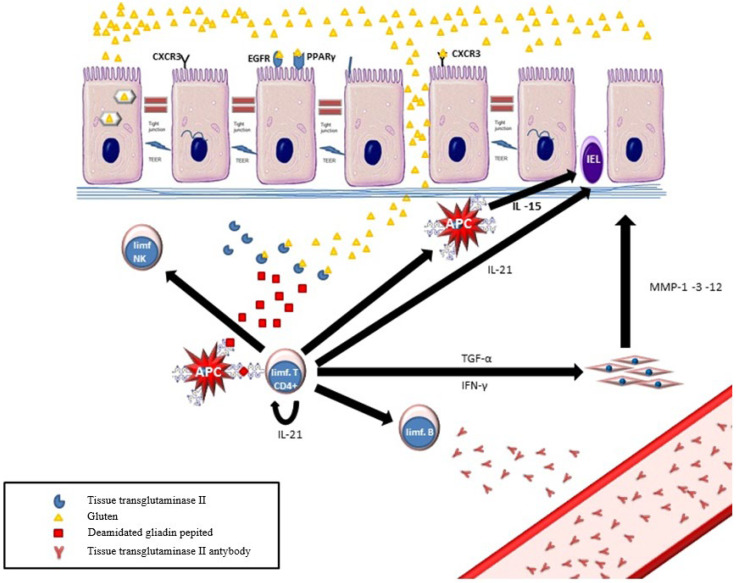
Schematic diagram of the basic immune reactions leading to the development of celiac disease. APC—antigen-presenting cell. TGF-α—transforming growth factor, α IFN-γ—interferon γ, TEER—transepithelial electrical resistance, PPARγ—Peroxisome proliferator-activated receptors, CXCR3—chemokine receptor, 3MMP—matrix metalloproteinases, and EGFR—epidermal growth factor receptor.

As it results from the above description, during a complex immune reaction, most cells involved in inflammatory processes are activated, both in terms of cellular and humoral responses (Figure 2). The above-mentioned prolamins, adequately to the HLA epitopes present, contribute to CD development in a complex mechanism led to direct the apoptosis of enterocytes, which leads to gradual villous atrophy. On the other hand, gliadin induces an increased production of EGF, which leads to cell hyperproliferation within intestinal crypts. The pathogenesis of celiac disease is extremely complex and still not fully elucidated yet.

### 1.4. Diagnosis

**Figure 3:**
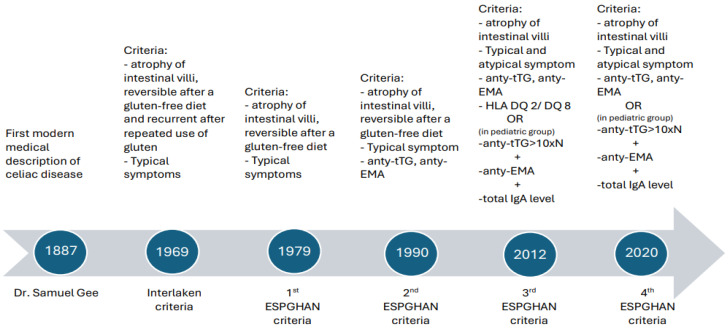
Changes in the diagnostic criteria for celiac disease over the years.

Knowledge about the pathogenesis of celiac disease has deepened over recent decades. As a result, the criteria for diagnosing celiac disease have changed over the years (Figure 3). According to the 2012 ESPGHAN (European Society for Pediatric Gastroenterology, Hepatology and Nutrition) criteria, diagnosis is based on the clinical picture (both typical and atypical symptoms) and intestinal villus atrophy type 2 or 3 according to the Marsh scale. Biopsy is recommended at upper G.I. endoscopy according to the following scheme—two samples from the duodenal bulb and four from the descending duodenum. In addition, the detection of specific anti-endomysial antibodies, anti-tissue transglutaminase 2 (anti-tTG), or anti-deamidated gliadin peptides (anti-DGP) at a concentration exceeding the norm by at least three times should be noted, as well as an elevated level of endomysial antibodies (EMAs) and the presence of the specific histocompatibility antigens HLA-DQ2 or DQ8. The sensitivity and specificity of serum antibody tests for celiac disease according to the different sources are presented in Table 3 [160,161,162].

Avoiding biopsy in pediatric patients who have levels of IgA anti-tTG antibodies ten times higher than the upper limit of normal, a high level of EMA, and fulfil the genetic criteria has been considered [163]. However, the 2020 modification of the above guidelines places the main emphasis on the determination of IgA anti-tTG antibodies and on the determination of the total IgA level. The determination of total IgA is necessary due to its frequent deficiency in CD patients. As a result, low levels of IgA anti-tTG antibodies may result from total IgA deficiency and produce a false-negative result. In the case of children and adolescents, the simultaneous finding of a ten times increase in the upper limit of IgA anti-tTG antibodies with a normal level of total IgA and a typical clinical picture allows for the diagnosis of celiac disease without the need for duodenal histology. It should be noted that IgA anti-tTG antibodies are also useful in the detection of gluten free-diet non-adherence in patients with established CD.

In adults, the presence of typical histological changes is necessary for CD diagnosis. Genetic tests assessing the presence of HLA-DQ2 and DQ8 antigens are used to exclude celiac disease in subjects observing a gluten-free diet. Nevertheless, HLA-DQ2/DQ8 has a limited role in the diagnosis of CD. This role is based on a negative predictive value in order to rule out CD in patients who are seronegative with typical histologic changes, in patients seronegative at the time of diagnosis, and in those patients with previously diagnosed CD before the introduction of celiac-specific serology [2].

These diagnostic criteria are also recommended by the American College of Gastroenterology (ACG). The ACG devotes part of its guidelines to intestinal permeability tests. Permeability tests are not recommended for the diagnosis of CD. They might be useful for the detection of gross changes in intestinal permeability associated with intestinal inflammation, but their sensitivity and specificity are too low for the diagnosis of CD [1,61].

### 1.5. New Diagnostic Techniques for Celiac Disease

New diagnostic techniques are still being sought to enable diagnosis with a greater precision. Recent studies indicate a higher reliability of the determination of antibodies against neo-epitope tTG complexed to gliadin (98–100% sensitivity and 93–96% specificity) in comparison with the assessment of anti-tissue transglutaminase antibodies (sensitivity: 74–100%, specificity: 78–100%) [160,161,162,164,165,166,167,168].

An additional test confirming celiac disease in the future may be the determination of the presence of T cells’ response to HLA-DQ2–α-gliadin complexes. A positive correlation was demonstrated between the number of gluten-reactive T cells in duodenal biopsy and histological damage in the course of celiac disease, as well as the concentration of anti-tissue transglutaminase antibodies [169]. It has been shown that three days of ingestion of gluten-containing food renders the memory T lymphocytes to be reactive against gliadin from gut-associated lymphoid tissue (GALT) and be detected in the peripheral blood of CD patients. These antigen-specific T-cells can be detected with enzyme-linked immunospot (ELISPOT) assays or by flow cytometry tetramer technology. Moreover, studies have been conducted on T cells collected from the peripheral blood of patients for the presence of the histocompatibility antigen HLA-DQ2 [170,171,172]. In the future, this test may become not only a new diagnostic method for celiac disease detection, but also a test to confirm the diagnosis of celiac disease in patients already observing a gluten-free diet, without exposing them to a long-term gluten challenge. The recent popularity of self-administered gluten-free diets without a clear indication represents a frequent challenge for clinicians when comes to the CD confirmation or exclusion. Analysis of the presence of gluten-reactive T cells in peripheral blood can also be used to assess adherence to a gluten-free diet [170,171,172,173]. Such a test could be helpful in diagnosing celiac disease, especially since current studies in healthy individuals with HLA-DQ 2.5+ have not shown any reactivity of memory T cells specific for immunodominant gluten epitopes [174]. Although Özgenel et al. and Cecilio et al. showed an increased frequency of HLA-DQ2/DQ8 in first-degree relatives of celiac patients [175,176], their use in the primary diagnosis of celiac disease is not confirmed [177].

New genetic determinants of celiac disease are still being sought due to the significant genotypic–phenotypic divergence among individuals with HLA DQ2/DQ8 antigens. A GWAS (genome-wide association study) study conducted in 336 celiac patients from Poland demonstrated a significant association between the development of celiac disease and the presence of the MSH5 gene [178]. In a study evaluating single-nucleotide polymorphisms (SNPs), 57 non-HLA variants predisposing to the development of CD were identified. In turn, within HLA, a significant predictive value was demonstrated for the presence of HLA-DQ 2.5 rs2187668, HLA-DQ7 rs4639334, and DQ8 rs7454108 [179,180,181,182]. So far, few studies have been published examining the association of non-HLA genes with the risk of developing and subsequent severity of CD.

In some patients with celiac disease, despite gluten-free diet adherence, the intestinal villi do not recover and chronic symptoms do not subside. In order to monitor and detect a group of patients who may require more careful surveillance and the introduction of additional management, it may be useful to detect patients’ whole-blood IL-2 release [183,184]. This relationship was confirmed by Tye-Din et al. in a study of 295 patients on a gluten-free diet who were challenged with gluten [185]. Gliadin-specific T cells found both in the G.I. tract [134,186,187] and selected from the peripheral blood [137,188] can also be used to assess unconscious exposure to ingested gluten. Zühlke et al. demonstrated an increased expression of CD38 on gluten-specific CD4+ T cells in patients after gliadin exposure. The study was conducted using blood samples from patients who underwent a gluten challenge test. Samples were incubated with an equal mixture of HLA-DQ2.5:gluten tetramers and were stained with the following antibody mixture: CD38-FITC, CD45RA-PE-Cy7. The activation status of tetramer + β7 + T_EM_ cells was assessed by the percentage of cell surface expression of CD38 on tetramer  +  β7  +  T_EM_ cells [189]. These tests can be used in the future to monitor gluten free diet adherence.

### 1.6. Treatment

According to the current guidelines, the basic method for treating celiac disease is an elimination diet (i.e., excluding products that may contain gluten) [1,2], i.e., foods produced using substrates derived from wheat, rye, and barley [61]. There is a lot of controversy about the use of oats in CD. On the one hand, they can enrich the diet with whole grains and nutritious dishes [190], but on the other hand, some studies show that they may contain traces of gluten [191]. It should be noted that the avenin present in oats does not have epitopes considered to be the source of the immunological reaction to gluten proteins in wheat, barley, and rye [192]. Current studies indicate that the consumption of 10 mg of gluten daily in patients with celiac disease should not cause an exacerbation of the disease, although in some cases, the daily dose may be several times higher [193,194,195,196]. The applicable certification standards allow for a gluten content of 20 ppm (20 mg per kilogram of product) in gluten-free products and 100 ppm in low-gluten products [197]. In the European Union, gluten-free products are marked with the crossed-out ear of wheat symbol and in accordance with “Commission Regulation (EU) 41/2009 on the composition and labelling of foodstuffs suitable for people intolerant to gluten”. The gluten content in food products marked with this logo may not exceed 20 mg per kilogram of product. A similar legal regulation was also introduced in the United States [198]. It should be noted that even trace amounts of gluten can lead to chronic inflammation in the intestinal mucosa [199,200].

In current times, considering that we encounter highly processed food on a daily basis, a gluten-free diet has become an extremely demanding form of treatment for patients. Patients must be aware of how to properly read food labels and follow the current product tests for gluten content. At the same time, a proper balance of the diet in terms of vitamins, minerals, and fiber should be taken into account [201]. This constant effort can affect quality of life [202]. In addition, gluten-free products, compared to those containing gluten, have a lower protein and fiber content, as well as nutritional value (i.e., lower intake of folate, iron, magnesium, selenium, calcium, and vitamins D, E, and some of group B [nestares, cardo]) compared to their counterparts containing gluten. Due to the consumption of a large amount of meat, a gluten-free diet is also a diet rich in saturated fats [203,204,205,206]. As a result, a poorly balanced gluten-free diet can lead to amino acid and protein deficiency, vitamin deficiency (especially vitamin A, B1, B6, B12, E, and D), electrolyte deficiency (primarily iron, zinc, calcium, and magnesium), and folic acid deficiency [207,208,209,210]. Moreover, gluten-free products are much more expensive than their gluten-containing counterparts, and some patients will be forced to limit their food intake or choose products of a lower quality, which, in many cases, may be an additional factor increasing such deficiencies [211,212,213].

However, in most cases, constant adherence to a gluten-free diet, especially among young patients, leads to complete recovery of the villi and resolution of the inflammatory infiltrate, despite the presence of trace amounts of gluten contamination in food [214]. In adults, especially over the age of 60, histological changes may not undergo complete remission despite strict adherence to a gluten-free diet [215,216].

It should be underlined that a gluten-free diet is connected to numerous economic, social, and psychological problems [202]. This is very expensive, and patients have to search for special stores with the adequate products, which are not uniformly localized. According to the studies conducted, trace amounts of gluten are currently detected even in naturally gluten-free products due to contamination resulting from production processes [217,218,219,220,221,222,223]. Even in products marked with the crossed-out ear of grain symbol, the safe gluten concentration is often exceeded [217,224,225,226]. The high quality of these products is not always warranted [211,212,213]. Gluten-free diet adherence poses social problems when visiting restaurants with friends, where gluten free food is less available [227,228,229]. In addition, this is connected with stigmatization, as being a person with special requirements can be traumatic, in particular for young people.

## 2. New Potential Treatment Strategies

### 2.1. The Use of Bacteria in the Treatment of Celiac Disease

The use of endopeptidases naturally produced by bacterial strains and fungi is one of the suggested treatment methods. *Flavobacterium meningosepticum* were the first strains in which the presence of endopeptidases capable of digesting prolamine-rich protein fragments was detected. As a result of gliadin degradation with endopeptidases, fragments are formed that are non-immunogenic for celiac patients [113]. Further studies have also shown the presence of similar endopeptidases in other bacteria and fungi [111,112]. Proteases have also been purified from the probiotic bacteria of *Lactococcus* and *Lactobacillus* [230,231,232]. Alpha-gliadins were reduced by more than 50% by peptidases produced by *Lactobacillus* spp. [233]. The endopeptidases described above have a very limited effect on gluten digestion. To achieve effective treatment, a fermentation process is necessary, which limits their use as an enzymatic supplement. Endopeptidases derived from *Lactobacillus* sp. are used in the fermentation process of flour, which can be used in further stages for bakery products [234,235,236,237]. Enzymes produced *by Lactococcus*, *Lactobacillus*, and *Flovabacterium* are not suitable for the oral supplementation of gluten-digesting enzymes. Further research in this area led to the discovery of endopeptidases that effectively break down gluten in the upper gastrointestinal tract, as described below.

### 2.2. Oral Supplementation of Endopeptidases

Clinical trials are currently underway on the oral administration of endopeptidase derived from Aspergillus Niger (AN-PEP). Although first reports indicate an effective reduction in the frequency of immune responses, the authors indicate that the dose of enzymes necessary to digest the gluten contained in food strictly depends on the type of meal and the method of its preparation, therefore, effective supplementation may prove difficult [238,239].

Oral preparations of endopeptidase mixture (ALV003) obtained from Sphingomonas capsulata (SC-PEP) and endopeptidase from barley seeds (EP-B2) have also been used in clinical trials. Studies conducted so far indicate that subjecting gluten-containing products to enzymatic treatment with ALV003 before consumption significantly reduces the immune response to prolamins contained in the meal [240,241,242]. Phase 2 clinical trials have shown that MGX003 reduced gluten-induced intestinal mucosal damage and symptom severity [243].

After a successful trial using 1.2 g of gluten per day for 6 weeks, which showed a significant decrease in the inflammatory response and a reduction in symptoms [243], further clinical trials with Latiglutenase were initiated in a large group of patients. Research has also been initiated on a computer-modified enzyme, Kuma 030 obtained from Alicyclobacillus sendaiensis, which is an endopeptidase that effectively degrades the linkage between proline and glutamine (TAK-062) [244]. In preliminary studies, it has shown a high efficiency in digesting a significant amount of gluten [245].

### 2.3. Modification of Immune Response

Apart from the application of bacterial and fungal endopeptidases, research is still ongoing on probiotic strains that reduce the inflammatory process. Strains of the genus *Bifidobacterium* spp. are mainly used in such studies. They were shown to reduce the level of TNF α, the number of IELs, and the level of antibodies in patients with celiac disease compared to a group of patients with celiac disease who did not receive probiotics [246,247,248,249].

Systemic steroid therapy has been used for years to modify the inflammatory response. Its possible use in celiac disease, especially if resistant to diet, has been considered for many years [250]. Studies using prednisolone at a dose of 1 mg/kg bw did not demonstrate any significant effect on villous regeneration [251]. However, the administration of 9 mg of budesonide in patients with refractory celiac disease for 3 months resulted in improvement and led to villous regeneration [252]. Numerous studies have indicated the effectiveness of budesonide in patients with celiac disease not responding to a gluten-free diet (NRCD), as well as refractory celiac disease (RCD) [253,254,255]. Azathioprine is also used in RCD. Remissions have been demonstrated in small groups of patients with this type of CD [256]. In subsequent studies, the use of azathioprine was found to be effective in type I RCD, but in some cases of type II RCD, its effect seems to be unsatisfactory [257,258]

There are very few case reports of the successful use of anti-TNFα antibodies in RCD. However, so far, there has been no broader analysis of such treatment. Therefore, such management should be considered as non-standard and limited to selected cases [259,260,261].

The monoclonal anti-IL-15 antibody (AMG 714; currently PRV-015) has been evaluated in CD [262,263,264]. In one study, the authors did not demonstrate any significant difference between the use of the preparation and a placebo in terms of pathological changes in the intestinal mucosa in patients exposed to a gluten challenge, but they did demonstrate a significant reduction in symptoms [265]. In another phase 2a study in a group of 28 patients, no effect on the course of the inflammatory process was demonstrated, but a reduction in symptoms was confirmed compared to the group receiving placebo [266]. A phase 2b clinical study (NCT04424927) is currently underway in adult patients with refractory celiac disease.

In vitro studies have shown that tofacitinib, a Janus kinase inhibitor, has the potential to regulate the activity of abnormal IEL cell populations. In a phase 2 open-label clinical study [(EudraCT): 2018-001678-10] in patients with RCD type 2, 12-week treatment with tofacitinib led to the resolution of diarrhea/loose stools and disappearance of abdominal pain and weight gain, however, the primary immunologic end point of an absolute decrease in total IELs was not met and mucosal improvement as a secondary end point was observed in four of six patients. In all patients, a rapid recurrence of symptoms, including weight loss, was observed after treatment discontinuation, while the reintroduction of therapy led to a rapid and complete improvement [267].

According to current knowledge, celiac disease is a Th1-mediated autoimmune process. Attempts are being made to modulate this response by redirecting patients’ immune response to a Th2-mediated pathway. For this purpose, CD patients received hookworm larvae (Necator americanus) transcutaneously. In the studies conducted so far, in small groups, patients undergoing the procedure developed gluten tolerance without other clinical implications [268,269,270]. Moreover, N. americanus infection in gluten-challenged patients leads to an increased microbial richness by improving homeostasis, which may normalize inflammatory parameters and increase gluten tolerance [271,272]. Although a study conducted in 54 patients with celiac disease showed that hookworm infection reduces symptoms after gluten ingestion in patients with celiac disease, it does not restore tolerance to long-term moderate gluten consumption (2 g/day) [273].

A polymer conjugated to a deamidated gliadin peptide (KAN-101) has also been developed, which, when administered intravenously, liver-targeted, is expected to induce immune tolerance to gluten. The first-in-human study of KAN-101 demonstrated an acceptable safety profile in patients with celiac disease. Furthermore, KAN-101 showed the potential to induce gluten tolerance by blunting the inflammatory response of gliadin-specific CD4+ cells and intestinal CD8+ cells after gluten challenge (NCT04248855) [274]. KAN-101 is currently being evaluated in phase Ib/II and phase II studies (NCT05574010, NCT06001177). Another similar strategy is the use of nanoparticles as a copolymer of gluten particles and PLGA (TAK-101). Currently, the second phase of clinical studies has been completed, confirming the safety of this preparation and demonstrating the lack of an immune reaction to 14-day gluten challenge—TAK-101 was well-tolerated in celiac patients and no evidence of systemic immunosuppression was observed (NCT03486990 and NCT03738475) [275]. A phase II study is currently underway to investigate the efficacy and safety of TAK-101 in preventing gluten-specific T cell activation in celiac patients on a gluten-free diet (NCT04530123).

There has also been an attempt to create a vaccine (Nexvax2) designed to induce gluten tolerance by modifying the T cell response. Clinical trials have been initiated in this aspect. After vaccination, the immune response to gliadin was significantly lower than in unvaccinated patients. Studies have confirmed lower concentrations of IL-2 and INF-γ, as well as significantly lower CD4+ T cells proliferation [276,277,278,279]. Although the initial phase 2 study by Hardy et al. showed a reduction in the immune response to gluten in the group receiving nexvax2 [279], a further study in 178 patients with celiac disease, including an analysis of symptoms following gluten ingestion, did not show a reduction in symptoms in the group receiving the vaccine compared to the group receiving a placebo [280].

### 2.4. Zonulin Inhibitors

In celiac disease, according to the pathophysiology described above, the contact of gluten with the intestinal mucosa results in an increase in zonulin release. This leads to enterocyte tight junction dysfunction and increased mucosal permeability [127,281,282,283]. Knowledge of this pathomechanism was used to develop a protein substance (larazotide acetate) with properties that regulate the tight junctions between enterocytes, modulate intercellular tension (TEER), and inhibit the zonulin effect. This leads to a reduction in the permeability of partially digested gliadin fragments and, thus, a reduction in the immune response. Additionally, larazotide promotes the repair of enterocyte structural defects resulting from direct reaction with gliadin [284,285,286,287,288]. Developed by 9 Meters Biopharma, it was investigated as an adjunctive treatment for celiac disease patients who continued to have symptoms despite adherence to a gluten-free diet. The trial was discontinued in 2022 after an interim analysis explaining that the additional number of patients needed to determine a significant clinical outcome between the placebo and larazotide was too large to support trial continuation [289]. The therapeutic potential of larazotide acetate was assessed to be lower than expected due to the presence of both paracellular and transcellular gliadin transport pathways, whereas larazotide acetate is intended to block only the paracellular pathway. On the other hand, the study conducted studies on the use of AT-1001, which is an inhibitor of paracellular permeability derived from a protein secreted by Vibrio cholerae, and found that it ameliorated the impaired intestinal permeability [290].

### 2.5. Tissue Transglutaminase 2 Inhibitors

A number of substances have been developed as tissue transglutaminase 2 (tTG2) inhibitors [291,292,293]. However, studies in mice have shown that a complete congenital deficiency of tissue transglutaminase 2 leads to numerous complications, such as glomerulonephritis, splenomegaly, and impaired phagocytosis [294,295]. For this reason, it is impossible to introduce complete transglutaminase 2 inhibition in clinical practice. There are ongoing studies investigating the use of partial tTG2 inhibitors in patients with celiac disease [296,297]. A phase II clinical trial of a selective oral inhibitor of activated tissue transglutaminase 2, ZED 1227, has been completed. In the initial phases of the study, ZED 1227 was shown to be effective in preventing gliadin deamidation. The application of the preparation in a group of CD patients undergoing gluten challenge also demonstrated good results, including a reduction in mucosal damage compared to the group receiving placebo [298,299]. A phase IIb trial is currently ongoing in CD patients experiencing symptoms despite following a gluten-free diet (EudraCT 2020-004612-97).

## 3. Conclusions

Celiac disease results from a complex immune reaction to gluten. The gradually expanding knowledge about its pathogenesis enables the development of new therapeutic strategies both in patients with a mild course of the disease and in those who do not observe clinical improvement after following a gluten-free diet. However, currently the only recognized treatment for celiac disease remains a gluten-free diet. The main clinical challenges are diet-refractory disease and the increased risk of small intestine neoplasia, which is particularly difficult to detect. Early small intestine cancers symptoms are not characteristic and diagnostic methods such as MR enterography and enteroscopy are not widely available and highly operator-dependent, although it is possible that, in the coming years, new diagnostic and treatment methods will also find their application in clinical practice.

## Figures and Tables

**Table 1:** Analysis of studies evaluating osteopenia and osteoporosis among patients with celiac disease. n/c—no data at that point.

Author	Years of Study	Country	Age	No. of Participation	Sex	Osteoporosis Frequency
Zanchetta et al. [23,25]	2011–2015	Argentina	Adults	30	Women	n/c
Bai et al. [21]	n/c	Argentina	Adults	25	Women	n/c
Galli et al. [29]	2010–2021	Itali	Adults	291	Women–men 3:1	Osteopenia + osteoporosis 63.5%
Ganji et al. [30]	Review of 56 article tile 2018	UK, Brazil, India, Hungary, Poland	Adults		Women–men 3:1	14.4%
Ganji et al. [31]	2014–2019	Iran	Adults under 55	387	Women–men 3:1	16.4%
Walker et al. [32]		USA	Adults	721	Women–men 3:1	19.6%
Sayar et al. [33]	2010–2019	Turkey	Adults under 50	84	Women and men	15.2%
Soracka et al. [34]		Poland	Adults under 50	30	Women	13.3%

**Table 2:** Prevalence of celiac disease in population studies across the world.

Country/Region	Years of Study	Studied Population	Test Used for Screening	Studied Population Number	Prevalence of CD
North America -USA [68]			Anty-EMA, anty-tTG, HLA DQ2/DQ8		
	1996–2001	At all ages		13,145	0.75%
South America [79]	2000–2013	Metanalyses of 72 seroprevalence studies, at all ages	Anty-tTG, anty-EMA		0.46–0.64%
Europe [73]	1991–2012	Metanalyses of 49 seroprevalence studies, at all age	Anty-tTG		1.3%
-Finland [64,71]	1994	Schoolchildren	Anty-EMA, anty-tTG	3645	1.5%
	2000–2001	Adults (30–93 years old)	Anty-EMA, anty-tTG, biopsies	6403	2.4%
-German [71]	1990, 2001	Adults (25–74 years old)	Anty-EMA, anty-tTG, biopsies	9201	0.3%
-Italy [71]	2000–2002	At all ages	Anty-EMA, anty-tTG, biopsies	7427	0.7%
-Russian [80]	1997–2001	At all ages	Anty-EMA, anty-tTG, biopsies	11,070	0.2–0.57%
Asia [66]		Metanalyses of 19 seroprevalence studies, at all ages	Anty-tTG, anty-EMA		1.6%
-China [81]		Metanalyses of 18 seroprevalence studies, at all age	Anty-tTG, anty-DGP		0.56%
-India [82]	1994–1995	Seroprevalence at all ages	Anty-tTG	10,488	1.44%
-Australia [83]	2007–2015	Seroprevalence at all ages	Anty-tTG	3011	1.56%
-Iran [84]		Seroprevalence, schoolchildren	Anty-tTG	1500	2%
-Saudi Arabia [85]		Healthy adults aged 20–60 years	Anty-EMA, anty-tTG, biopsies	980	1.9%
-Israel [86]	2012–2013	Seroprevalence at all ages	Anty-tTG	403,283	1.56%
Africa:					
-Tunisia [87]		Adults (blood donors)	Anty-EMA	2500	0.28%
-Egypt [88]		Children	Anty-tTG	1500	0.53%
-Saharawi population [89]		At all ages	Anty-tTG, anty-EMA	975	5.6%

**Table 3:** Sensitivity and specificity of serum antibodies tests for celiac disease [160,161,162].

Antibody	Sensitivity (%)	Specificity (%)
Anti-tTG IgA	63–96.8	91.0–100
EMA IgA	80–93.7	95–100
DGP IgG	18–84.4	98.5

## Data Availability

No new data were created or analyzed in this study. Data sharing is not applicable to this article.
